# Initial clinical radiological findings and staging to predict prognosis of primary hepatic angiosarcoma: A retrospective analysis

**DOI:** 10.1371/journal.pone.0225043

**Published:** 2019-11-11

**Authors:** Wei-Hsin Yuan, Anna Fen-Yau Li, Hui-Chen Hsu, Yong-Sin Hu, Rheun-Chuan Lee

**Affiliations:** 1 Division of Radiology, Taipei Municipal Gan-Dau Hospital (managed by Taipei Veterans General Hospital), Taipei, Taiwan, Republic of China; 2 School of Medicine, National Yang Ming University, Taipei, Taiwan, Republic of China; 3 Department of Radiology, Taipei Veterans General Hospital, Taipei, Taiwan, Republic of China; 4 Department of Pathology, Taipei Veterans General Hospital, Taipei, Taiwan, Republic of China; 5 Department of Medical Imaging, Taiwan Adventist Hospital, Taipei, Taiwan, Republic of China; National Institutes of Health, UNITED STATES

## Abstract

**Objective:**

Primary hepatic angiosarcoma (PHA) is extremely rare and most patients die within 12 months of diagnosis. The object of the study is to determine the association of initial clinical-radiological features and staging with outcomes in patients with PHA.

**Methods:**

The medical records of adult patients with PHA were retrieved from an electronic medical record database and a pathology database and retrospectively reviewed. During 10 years, 22 eligible patients were included. Data extracted focused on the information before the first formal treatment with a pathological proof, including demographic characteristics, medical history, laboratory data, preliminary images, histopathological records, treatment, and follow-up survival period. Two radiologists blindly re-analyzed preliminary images of all 22 patients together and recorded tumor features and imaging stage based on the American Joint Committee on Cancer (AJCC) 8th edition tumor-node-metastasis (TNM) Staging System for hepatocellular carcinoma. A radiologist compiled the initial clinical data and preliminary image stage to analyze the association with patients’ survival outcome.

**Results:**

Higher aspartate aminotransferase (AST), higher total bilirubin (TB), lower albumin (ALB), longer prothrombin time (PT) and lower platelet count of serum relative to the normal reference range were more common in patients who survived ≤ 90 days (all P < 0.05). Overall survival was much better in patients with single PHA than in those with other tumor patterns of multiple PHA (all P < 0.05). Overall survival determined by preliminary imaging showed significant differences between stage I and stage III (P = 0.044), stage I and stage IV (P = 0.011), and stage III and IV (P = 0.047). No patients were at stage II.

**Conclusions:**

Initial serum levels of ALT, TB, ALB, and PT, platelet count, single mass in liver, and preliminary imaging staging could help predict survival outcomes of patients with PHA.

## Introduction

Primary hepatic angiosarcoma (PHA) is very rare, accounting for 1–2% of primary hepatic malignancies [1, 2]. PHA has been related to hemochromatosis, anabolic steroid, neurofibromatosis type 1, and chronic exposure to arsenic, vinyl chloride, thorium dioxide, and radiation [3, 4], although the main causes or risk factors for PHA remain unknown [4].

PHA occurs mainly in men aged over 60 years old [5, 6]. Rapidly progressing malignancies and non-specific symptoms of PHA delay in examinations and treatments, which result in poor prognosis [7]. Histopathological examination can confirm PHA, [4, 8, 9]. In addition, CD31, CD34, and factor VIII-related antigen are often positive in the diagnosis of PHA [8].

When PHA is confined to one lobe of the liver without any metastatic lesions, a complete surgical resection is suggestive and may benefit prognosis [7, 8, 10]. Radiation therapy doesn’t work because PHA is radioresistant [4]. The efficacy of chemotherapeutic regimens seems limited [7–9]. Patients with PHA have a median survival of less than 6 months, even with treatments [4, 8]. Most patients die within 12 months of diagnosis [1]. However, few studies reported the prognosis of PHA patients according to clinical information, radiological findings and staging before the first formal treatment with a pathological proof. In the study of Kim H.R. et al [4], 3 (60%) of 5 PHA cases with an advanced stage survived less than 90 days with lower hemoglobin (Hb), lower platelet (PLT) and higher aspartate aminotransferase (AST) or/and higher alanine transaminase (ALT) relative to the normal reference range. Huang N.C. et al [3] compared 17 PHA patients with short- and long-term survival (11 patients < 2 years versus 6 patients ≥ 2 years) and found patients with long-term survival tended to have a smaller maximum tumor size (7.2±4.7 cm vs. 12.8±7.0 cm, P = 0.08) and metastasis (66.7% vs. 18.2%, P = 0.11). Huang I.H. et al [11] used the American Joint Committee on Cancer (AJCC) tumor-node-metastasis staging system for hepatocellular carcinoma to assess 34 patients with PHA and found that the overall survival of stages I and IVB was significantly different (P = 0.0182) but that of other two stages did not reach statistical significance (I vs II, P = 0.4743; I vs IIIA, P = 0.1487; II vs IIIA, P = 0.1531; II vs IVB, P = 0.0629; IIIA vs IVB, P = 0.9972).

Therefore, we retrospectively reviewed and reanalyzed the clinical features, radiologic findings, and radiologic staging before the first treatment of patients with histologically-proven PHA at our teaching hospital, Taipei Veterans General Hospital (Taipei, Taiwan), with the aim to determine the association between prognosis and initial clinical data and relate prognosis to preliminary imaging findings and staging.

## Materials and methods

### Patients

The study received an inform consent waiver from the Institutional Review Board of Taipei Veterans General Hospital (TVGH), which waived informed consent because of the retrospective cohort study.

A research doctor (YSH) blinded to the study hypothesis abstracted pathology results from the pathology database at TVGH using the key words “liver angiosarcoma” and “hepatic angiosarcoma.” From January 1, 2008 to December 31, 2017, 50 patients had records matching the search key words.

We included the patients who had PHA pathologically proved at TVGH. The exclusion criteria were as follows: primary diagnosis not hepatic angiosarcoma, age < 20 years, pregnant, no initial clinical and imaging data, and transfer to another hospital after treatment of PHA.

We excluded 18 of 50 patients in our series because they actually had epithelioid hemangioendothelioma of the liver. We also excluded the other ten of 50 patients with angiosarcomas actually not originating from liver as follows: one with angiosarcoma on the right arm, one on the left breast, one on the right thigh, one in the urinary bladder, two on scalps, one on cheek, one in the left supraclavicular area, one in the spleen and one in the mediastinum. The remaining 22 patients, who had PHA without any exclusion criteria, were included in this study.

### Clinical evaluation

The research doctor (YSH) retrieved the detailed clinical information of 22 cases from the hospital electronic medical record database, and focused on the data before the first formal treatment with a pathological proof, including demographic characteristics, medical history, initial laboratory and imaging tests, and histopathological records. Treatment modality and the prognosis of patients were also recorded. The patients’ demographic characteristics and medical history included the following: gender, age, chief presenting symptoms, history of smoking or drinking, history of hypertension and diabetes mellitus, malignancy history, liver cirrhosis, tumor rupture, markers of hepatitis B virus (hepatitis B surface antigen) and hepatitis C virus (anti-hepatitis C antibody) infection, and probable oncogenesis.

To evaluate patients with liver malignancy, the clinicians in TVGH ordered initial laboratory and image tests which were necessary for patients to serve as the guide of the first formal treatment with a pathological proof.

Initial imaging data of 22 patients for detecting PHA and metastases might include chest x-ray; abdominal x-ray; plain films of lumbar spine, thoracic spine, cervical spine skull or four limbs; abdominal sonogram; abdominal computed tomography (CT) and/or magnetic resonance imaging (MRI); whole-body bone scan; and whole-body positron emission tomography (PET)/ CT. Tumor rupture resulting in hemoperitoneum was determined by sono-guided aspiration, CT and/or MRI. Liver cirrhosis was certified by past histories and abdominal sonography, CT or MRI images. Initial laboratory data might include white blood cell count (WBC), hemoglobin (Hb), platelet count, albumin (ALB), alanine aminotransferase (ALT), aspartate aminotransferase (AST), total bilirubin (TB), carcinoembryonic antigen (CEA), carbohydrate antigen19–9 (CA 19–9), and alpha-fetoprotein (AFP).

### CT imaging techniques

Abdominal CT images, chest CT or brain CT images were obtained using a multiple detector computed tomography (MDCT) scanner. MDCT scanner of abdomen included LightSpeed VCT (64-slice, n = 2), LightSpeed QX/I (4-slice, n = 1), GE Healthcare; Emotion 16 (16-slice, n = 1), Somatom Sensation 16 (16-slice, n = 2), Siemens Healthcare; Brilliance Big Bore (16-slice, n = 1), Brilliance 40 (40-slice, n = 1), Brilliance 64 (64-slice, n = 7), iCT 256 (256-slice, n = 1), Philips Health-care. Abdominal CT scans were obtained without and/or with intravenous iodine-based contrast agent, which included Iopamidol (Iopamiro 370^®^; Bracco s.p.a., Milan, Italy, 370 mg iodine [I]/ml) in 9 patient, Iobitridol (Xenetix 350^®^; Guerbet, Aulnary-sous-Bios, France, 350 mg I/ml) in 3 patients and Iohexol (Omnipaque 350^®^; GE healthcare, Co. Cork, Ireland, 350mg I/ml) in 2 patients. Fourteen of 22 patients received an intravenous power injection as a bolus of 1.2 ml/kg iodine-based contrast medium at 2–2.5 mL/second (s). Post-contrast CT images with a dynamic protocol was acquired during hepatic arterial phase (25 s after the start of intravenous contrast medium [CM] injection), portal venous phase (approximate 70 s after CM injection) and, delayed phase (approximate 180 s after CM injection). Of 22 patients, 2 patients received only pre-contrast brain CT, 1 patient did both pre- and post-contrast brain CT, 3 patients had only post-contrast Chest CT, and 1 patient only pre-contrast chest CT. Post-contrast brain and chest CT images were obtained after completing intravenous injection of iodine-based contrast medium, whose volume was approximately 60–80 ml and rate was 1ml/s. The dataset was reconstructed with slice thickness for image viewing of axial and coronal images was 5mm using each type of MDCT scanner. The reconstruction matrix for MDCT scans was 512 × 512.

### MR imaging techniques

Thirteen of the 22 patients received variabilities in abdominal MRI techniques and sequences. Abdominal MR images with or without intravenous gadolinium (Gd)-based contrast medium were obtained using a 1.5 or 3 Tesla (T) MRI scanner, which included Magnetom Symphony (1.5T, n = 1), Siemens Healthcare; Signa HDxt (1.5T, n = 8), Optima MR450w (1.5T, n = 1), Genesis Signa (1.5T, n = 1), Discovery MR750 (3.0T, n = 1), Signa Excite (1.5T, n = 1) GE Healthcare. Thirteen of the 22 patients underwent pre-contrast MRI protocols, which included 2-dimentinal (2D) and 3-dimentional (3D) sequences as follows: 2D T1-weighted (T1W) gradient-echo (GRE) in-phase images (axial section, Time of repetition [TR]/ time of echo [TE] = 80–185 milliseconds [ms]/4.2–5.8 ms, matrix = 256–320x134–224, flip angle = 70°-80°, field of view (FOV) = 320–420, slice thickness [ST] = 6–9 millimeter [mm]), 2D T1W out-of-phase images (axial section, TR/TE = 80–185 ms /1.8–2.5 ms, matrix = 256–320x134–224, flip angle = 70°-80°, FOV = 320–420, ST = 6–9 mm), 2D fat-suppressed (FS) T2-weighted (T2W) fast spin echo (FSE) images (axial section, TR/TE = 2400 ms-18000 ms /77.95 ms-103.9 ms, matrix = 256–320x128–320, flip angle = 90°, FOV = 320–420, ST = 6 to 9 mm), 3D FS T1W GRE images (axial and coronal sections, TR/TE = 3.12 to 6.21 ms/0.83 to 3.13 ms, matrix = 256–332x106–224, flip angle = 9°-15°, FOV = 320–420, ST = 3.6–5 mm), 2D diffusion-weighted images (DWI, b value = 800) and apparent diffusion coefficient (ADC) (axial section, TR/TE = 5000–12857.1 ms/57.5–83 ms, matrix = 120–128x96–192, flip angle = 90°, FOV = 320–420, ST = 8 or 9 mm). However, 2 of the 13 patients received neither DWI nor ADC sequences. One patient only received pre-contrast MRI but twelve of the 13 patients received post-contrast sequences of abdominal MRI with a dynamic MRI study. Intravenous Gd-based contrast medium included gadobenate dimeglumine (MultiHance^®^; Bracco s.p.a., Milan, Italy, 0.5mmol/ml) used in one patient, gadodiamide (Omniscan^®^; GE Healthcare, Ireland Limited CorK, Ireland, 0.5mmol/ml) in 2 patients, gadoterate meglumine (Dotarem^®^; Guerbet, Aulnay-Sous-Bios, France, 0.5mmol/ml) in 3 patients and gadobutrol (Gadovist^®^; Bayer AG, Berlin, Germany, 1mmol/ml) in 6 patients. Twelve of the 22 patients were administrated by a power injection as a bolus of 0.1mmol/kg gadolinium chelate at 2 mL/s, followed by a flush of 20 ml saline at the same injection rate. Post-contrast dynamic MRI was acquired at 25 seconds (hepatic arterial phase) following an intravenous gadolinium contrast medium injection, then again at approximately 70 seconds (portal venous phase) and at 3 minutes (delayed phase). Post-contrast protocols included 2D dynamic contrast-enhanced FS T1WI (axial sections, TR/TE = 100–210 ms/1.8–3.5 ms, matrix = 256–320x192–224, flip angle = 80° or 90°, FOV = 320–48, ST = 6–9 mm) in one patient and 3D dynamic FS T1WI (axial and coronal sections, TR/TE = 3.12–6.21 ms /0.83–3.13 ms, matrix = 256–332x106–224, flip angle = 9°-15°, FOV = 320–440, ST = 3.6–7 mm) in 11 patients.

### Analysis of images and pathological diagnosis

Two experienced radiologists (HCH, with 20 years of experience; and RCL, with 30 years of experience), without knowledge of the histopathological results, re-analyzed the initial images of all 22 patients together on a picture archiving and communication system monitor. Final results were decided by consensus and recorded.

Biopsy for each lesion in liver could result in lethal complications to PHA patients but imaging studies are helpful for diagnosis [1, 4]. In re-analyzing images, we compared the attenuation of tumor and intra-tumoral foci on pre-contrast CT scans of abdomen with the normal liver parenchyma and graded as higher than, lower than or equal to the attenuation of adjacent normal liver parenchyma. A thin-walled well-circumscribed homogeneous water-attenuation cyst (0–20 Hounsfield unit [HU]) in liver was identified as simple cyst on pre-contrast CT scans [12, 13] unless the cyst showed some enhancement on post-contrast CT images. On MRI, the signal intensity characteristics of tumors was compared with normal liver parenchyma. On post-contrast CT and MRI, enhancement pattern of liver tumors was classified as nodular, bizarre-shaped or ring enhancement. Enhancement location of tumor was characterized as peripheral or intra-tumoral regions of neoplasm.

Whole-body bone scans and PET/CT were used to detect distant metastasis, which showed avid uptake of 99mTc-methylene diphosphonate and fluorine-18 fludeoxyglucose, respectively [14]. Multiple differently sized shaped nodules and ground-glass opacities or multiple thin- walled cysts at bilateral lungs present on chest x ray or chest CT scans indicated lung metastases [15]. Metastases were identified on CT or MR images when space-occupying lesions at extrahepatic organs showed similar PHA features on CT or MRI. Expansile osteolytic lesion at bony structure on plain film or CT scans indicated bone metastases.

The recorded features of tumors on CT and/or MR images included: liver cirrhosis, tumor pattern, location and maximum size of hepatic tumors, tumor density and intra-tumoral hyper-attenuation hemorrhage [16, 17] on CT scans, signal intensity on MRI, contrast enhancement on CT and/or MRI, metastatic site, tumor-node-metastasis (TNM) classification, and preliminary imaging stages. When an irregular surface of the liver appeared on abdominal sonography, abdominal CT and/or MRI, the liver was classified as cirrhosis. The preliminary imaging stage was based on the American Joint Committee on Cancer (AJCC) TNM staging system, 8^th^ edition. Due to the location of PHA in the liver, tumor invasion into hepatic vessels, the presence of multiple tumors and regional lymph nodes metastases, the TNM staging system for hepatocellular carcinoma was used to evaluate PHA staging [11]. Lymph node metastasis on CT or MRI was defined as lymph nodes with a long axis greater than 1.5 cm and a short axis greater than 1 cm [18, 19]. An experienced pathologist (AFYL) with 27 years of experience in liver pathology reviewed and confirmed the pathological diagnosis of 22 patients with PHA.

### Follow-up and assessments

Overall survival (OS) period defined as the time from the PHA diagnosis to death from any cause or last follow-up visit. Follow up of this study ended on February 23, 2018. A fourth radiologist (WHY) integrated the data of all patients to analyze the initial clinical and image characteristics, and their association with survival.

### Statistical analysis

Most PHA patients die within 12 months of diagnosis [1]. To identify the factors affecting length of survival, we compared patients with short- (the first quartile survival) and long-term survival (≤ 90 versus > 90 days). Because of the small number of patients, the study applied nonparametric tests (Mann-Whitney U test) for continuous variables and the χ2 or Fisher’s exact test for categorical variables to assess differences between characteristics in the subgroups. Survival analysis proceeded with the Kaplan-Meier method and the log-rank test. The study used SPSS software version 19 (SPSS Inc., Chicago, IL) for all statistical analyses with the significance level set at P < 0.05.

## Results

### Baseline medical characteristics

Table 1 describes the baseline medical characteristics of the 22 patients. The median age of all patients was 68 years (range, 38–83 years; mean age, 66.5 years). There were 15 male (68%) and seven female (32%) patients. Fifteen (68%) of the 22 patients displayed the symptoms/signs associated with liver or gastro-intestinal disease. Most patients (n = 11, 50%) presented with upper abdominal pain or discomfort. Seven patients (7/22, 32%) had symptoms/signs non-specific to abdominal disease. None of the 22 patients had a significant exposure history to specific carcinogens related to PHA. Six patients (27%) drank every day; six patients (27%), all male, smoked. Four patients (4/22, 18%) had a previous history of malignancy, including B cell lymphoma of the stomach in case 5, transitional cell carcinoma of the right kidney in case 6, colon carcinoma in case 16, and urothelial carcinoma of the urinary bladder in case 15. None of the 22 patients had any significant past medical or family history of PHA.

**Table 1:** The demographic information of 22 patients at the initial admission.

Case	Age/Sex	Chief complaint	Smoking/Drinking	Hepatitis	Liver cirrhosis	Tumor rupture	DM/HT	Previous malignancy
1	62/M	Abdominal fullness, general weakness	Y/Y	N	N	N	Y/Y	N
2	38/F	Abdominal fullness, poor appetite	N/N	B	N	N	N/N	N
3	65/M	Abdominal fullness	N/N	N	N	N	Y/Y	N
4	62/M	RUQ abdomen pain	N/N	N	N	N	Y/N	N
5	75/M	Right lower leg edema	Y/N	C	N	N	N/Y	Y
6	82/M	Cough with bloody sputum	Y/N	N	N	N	N/N	Y
7	52/M	hematuria	Y/Y	N	N	N	Y/Y	N
8	56/F	Coffee ground vomitus and tarry stool	N/N	N	Y	N	N/N	N
9	73/M	Acute abdominal pain, conscious change	Y/Y	N	N	Y	Y/Y	N
10	73/M	incidental findings,	N/N	B	Y	N	N/N	N
11	80/F	Sudden onset of abdominal pain	N/N	N	N	Y	N/N	N
12	61/M	Yellowish skin	N/N	N	N	N	Y/N	N
13	50/M	Abdominal pain	N/Y	N	N	N	Y/Y	N
14	66/M	Epigastric pain	N/N	N	N	N	N/Y	N
15	55/M	Upper abdominal dull pain	Y/Y	N	N	N	N/Y	Y
16	83/F	Intermittent hiccup, bowel habit change	N/N	N	N	N	Y/Y	Y
17	79/F	Progressive dyspnea, leg edema	N/N	N	N	N	N/N	N
18	78/M	Yellowing skin	N/Y	N	N	N	N/N	N
19	73/F	Abdominal distension	N/N	N	Y	N	Y/N	N
20	70/M	Physical examination found	N/N	N	N	N	Y/Y	N
21	72/F	Epigastric pain, easy skin bruising	N/N	N	N	N	N/N	N
22	58/M	Soreness of right shoulder	N/N	N	Y	N	N/N	N

M: male; F: female; DM: Diabetes mellitus; HT: Hypertension; Y: Yes; N: No; B: Hepatitis B; C: Hepatitis C; RUQ: right upper quadrant.

### Initial laboratory data

Table 2 shows the initial laboratory profiles of the patients enrolled in this study as well as the normal reference range of routine blood and laboratory tests (biochemistry tests) in our hospital. Not all of the 22 patients received all laboratory examinations. Ten patients had Hb < 10 g/dl. Case 9 and Case 11 had tumor rupture but Hb of both were > 10 g/dl. Nineteen patients (86%) had anemia and fourteen (64%) showed thrombocytopenia. Case 2 had the lowest Hb (4 g/dl) of all patients. No estimates of Child-Pugh grading were available in their charts.

**Table 2:** Initial laboratory data of 22 patients with primary hepatic angiosarcoma.

Case	WBC	Hb	PLT	ALB	TB	PT	ALT	AST	AFP	CEA	CA-199
1	11610	6.1	112000	3.4	2.31	11.7	64	86	-	-	-
2	7500	4	140000	3.2	1.46	14.1	17	47	18.32	0.78	8.86
3	6400	11.6	127000	4.5	0.74	10.8	14	19	-	-	-
4	11450	8	141000	4.2	0.46	11.4	12	34	8.78	2.55	10.65
5	8070	11.7	339000	4.5	0.49	10.4	16	32	2.2	2.47	8.17
6	7300	7.7	120000	3.3	-	13.4	13	23	2.16	1.37	11.59
7	9900	8.1	264000	3.8	0.21	10.4	17	14	5.07	1	3.14
8	7600	10.3	105000	3.1	26.07	18.2	15	48	5.31	2.7	2.0
9	6900	10.1	164000	4.2	0.67	11.6	22	25	1.02	1.8	9.1
10	4130	16.1	136000	3.6	1.12	10.7	35	65	2.45	22.4	17.3
11	5680	11.3	156000	3.4	0.68	10.4	13	37	-	-	-
12	6500	13.7	100000	3.3	4.83	14.6	29	24	1.93	-	-
13	8500	10.7	339000	3.4	1.24	11.3	46	30	5.71	2	3.3
14	6200	9.2	111000	-	2.83	14.5	41	104	7.05	1.2	5.6
15	4800	7.7	149000	3.8	0.35	11.2	46	41	4.35	-	20
16	8300	12.1	220000	3.9	0.44	10.6	22	23	2.12	3.6	7.8
17	7000	8	116000	3.2	2.67	12	75	174	2.38	4.2	80.5
18	4300	9.1	91000	-	8.71	14.3	64	68	2.69	3.6	6.8
19	5300	14.4	44000	4.1	2.6	15.4	31	28	4.22	2.6	17.2
20	5660	11	201000	4	0.39	10.4	26	16	3.27	4.8	32.1
21	4000	8.1	43000	2.9	2.81	14	51	80	1.64	3.5	4
22	7400	13.8	202000	3.4	0.98	11.1	34	34	4.51	2.4	5.5

Laboratory data and normal range; WBC: white blood cell, 4500-11000/cumm; Hb: hemoglobin, 14–18 g/dL (male), 12–16 g/dL (female); PLT: platelet, 150000-350000/cumm; ALT: Alanine transaminase, 0–40U/L; AST: aspartate transaminase, 5–45 U/L; TB: total bilirubin, 0.2–1.6 mg/dL; PT: prothrombin time, 9.4–12.5 sec; ALB: albumin, 3.7 to 5.3 g/dL; AFP: alpha-fetoprotein, 0–20.00ng/mL; CEA: carcinoembryonic antigen, 0.0–5.0 ng/dL; CA-199: carbohydrate antigen 19–9, 0.0–37.0 U/mL.

### Initial imaging findings and preliminary stage

Table 3 summarizes the initial imaging findings and stage before the first formal treatment with a pathological proof. According to the re-analyzing records of two radiologists in this study, all the 22 patients received chest x-rays and abdominal CT (n = 16) and/or MRI (n = 13); seven patients underwent both abdominal CT and MRI. Sixteen patients received abdominal sonogram, one received whole body PET/CT, three received whole body bone scans, three received brain CT, four had chest CT and 12 received L spine or abdominal x ray.

**Table 3:** Preliminary imaging features and stage, immunohistochemical staining results, treatment modality, and overall survival of 22 patients with primary hepatic angiosarcoma.

Case number	Tumor pattern/location of liver on CT, MR	Maximum size of tumor(cm) on CT/MR	TNM*	AJCC stage On images	Immuno-histology positive	Treatment modality	Survival (days)	Alive
1	MNDM/Bil	12.2	T3N0M1	IV	CD34	TAE	55	D
2	MN/Bil	4.6	T3N0M1	IV	CD34, CD31 hepatocyte	CTx	45	D
3	MN/Bil	5.7	T3N0M1	IV	CD31	Supportive care	5	D
4	SM/Rt	8.5	T1N0M0	I	CD31, FLi-1	One Segmentectomy +CTx+PEIT	555	D
5	SM/Rt	12.7	T1N0M0	I	CD31, FLi-1	Trisegmentectomy +CTx	211	C
6	MNDM/Bil	16.9	T3N0M1	IV	CD31, CD34, FLI-1, vWF	Supportive care	37	D
7	SM/Rt	10.3	T1N0M0	I	CD31, Fli-1, vWF	Bisegmentectomy+RFA+Bisegmentectomy	2078	C
8	MNDM/Bil	8	T3N0M0	III	CD31, CD34.	Supportive care	40	D
9	MNDM/Bil	10.7	T4N0M0	III	CD31, CD34	TACE	103	D
10	MNDM/Bil	9	T3N0M1	IV	CD31, CD34,	CTx	149	D
11	MNDM/Bil	13.6	T4N0M0	III	CD31, CD34	TAE+CTx+TACE+RTx	375	D
12	MN/Bil	5.7	T3N0M0	III	CD31. CD34, Fli-1	Supportive care	59	D
13	MNDM/Bil	12.5	T3N0M0	III	CD31, Fli-1	CTx+RTx	644	D
14	MNDM/Bil	22	T3N0M1	IV	CD31, CD34, Fli-1	TAE	26	D
15	MNDM/Bil	11	T3N0M0	III	ERG1, CD34, CD31	CTx+tumor resection +Y-90+TACE	989	D
16	MNDM/Rt	8.3	T3N0M0	III	ERG1, CD34, CD31	TACE+bisegmentectomy +CTx	256	D
17	MNDM/Bil	18.4	T3N0M0	III	ERG1, CD34, CD117	Supportive care	17	D
18	DIL/Bil	20	T3N0M0	III	ERG1, CD34	CTx	36	D
19	MNDM/Bil	7.6	T3N0M0	III	ERG1, CD31, CD 34	CTx, TAE	131	D
20	MNDM/Bil	7.4	T3N0M0	III	ERG1, CD31, CD34,	Y-90	198	D
21	DIL/Bil	23	T3N0M0	III	ERG1, CD31, CD34	CTx	34	D
22	DIL/Bil	14	T4N0M0	III	ERG1, CD31, CD34	TACE	110	D

MNDM, multiple nodules with a dominant mass; MN, multiple nodules; SM, single mass; DIL, diffuse infiltrating lesions; Bil, Bilateral lobes of liver; Rt, Rt lobe of liver; TAE, Trans-arterial embolization; TACE, Trans-arterial chemoembolization; PEIT, Percutaneous ethanol injection therapy; CTx, chemotherapy; RTx, Radiotherapy; Y-90, Yttrium-90; D, died; C, censored.

* The American Joint Committee on Cancer (AJCC) 8th TNM staging system for hepatocellular carcinoma is used to assess 22 patients.

**Fig 1:**
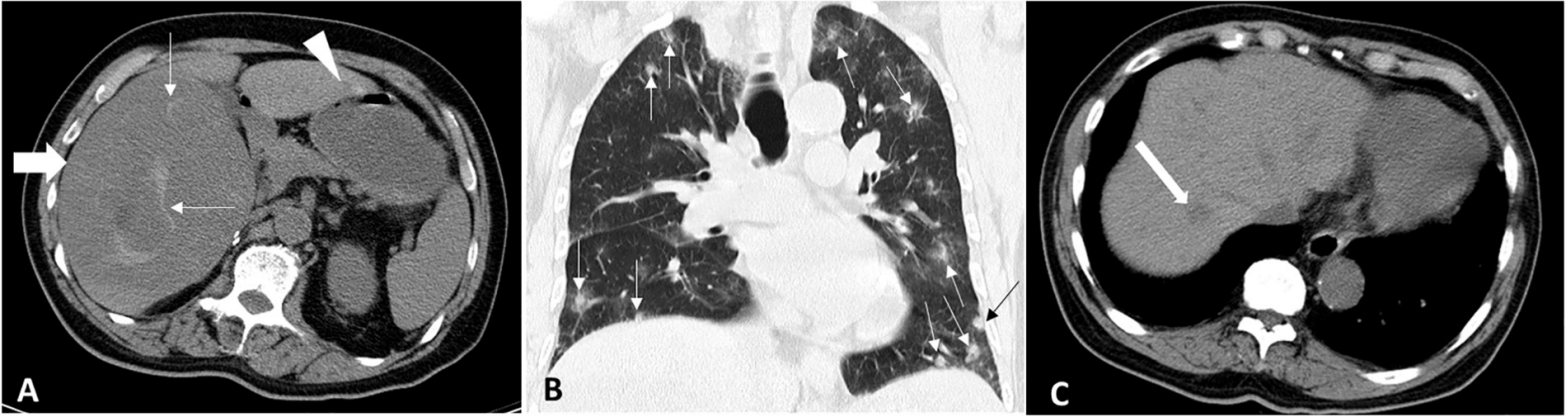
An 82-year-old man with primary hepatic angiosarcoma (case 6). (A) Transverse pre-contrast computed tomography (CT) image shows a dominant low dense mass (thick arrow) in the right lobe with small intra-tumoral high density foci (thin arrows), which suggest hemorrhage. Another smaller tumor in the left lobe of the liver is also noted (arrowhead). (B) Coronal pre-contrast CT image with lung window depicts multiple lung metastases (arrows) in the bilateral lungs. (C) Transverse pre-contrast CT image shows a small low density nodule (arrow) in the right lobe of liver. The mean value of CT Hounsfield unit (HU) for the nodule (arrow) is 39, which is greater than water attenuation (0–20 HU). This study classifies the tumor pattern of case 6 as multiple nodules with a dominant mass in liver.

**Fig 2:**
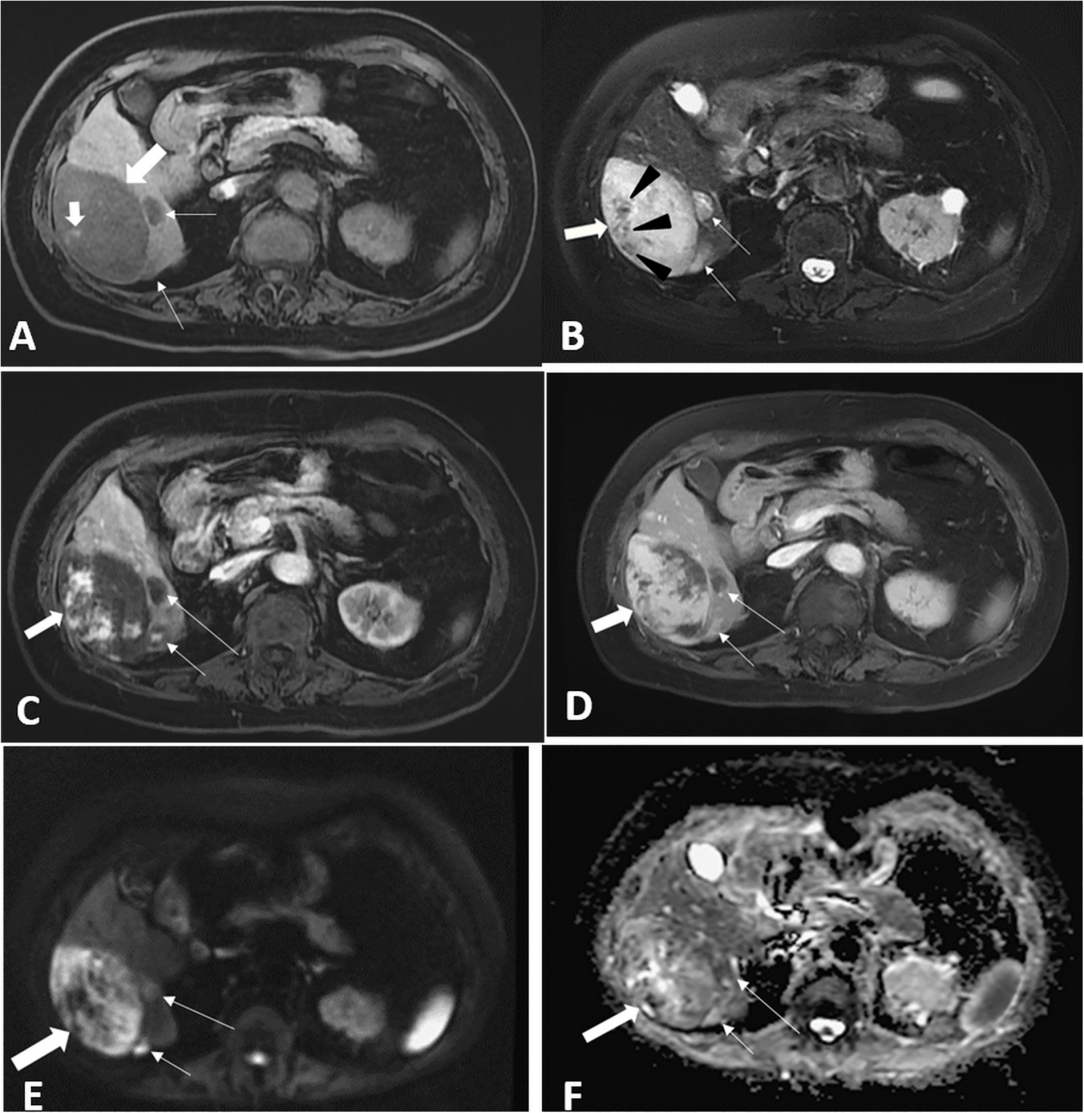
An 83-year-old woman with primary hepatic angiosarcoma (case 16). (A) Transverse 3D fat-suppressed T1-weighted gradient-echo pre-contrast (TR/TE = 6.10/3.13) magnetic resonance (MR) image shows a hypointense dominant tumor (thick large arrow) with a tiny focus of hyperintensity, which suggest hemorrhage (thick small arrow). Two smaller satellite tumors display hypo-intensity (thin arrows). (B) Transverse 2D fat-suppressed T2-weighted fast spin-echo pre-contrasted (TR/TE = 14000/97.664) MR image shows the dominant tumor (thick arrow) and two satellite nodules displaying heterogeneous hyperintensity (white arrows). Multiple hypo-intense foci within the dominant tumor are noted (black arrowheads). (C) Transverse fat-suppressed T1-weighted post-contrast image (TR/TE = 6.11/3.13) on the arterial phase shows bizarre-shaped enhancement patterns in the small satellite (short thin arrow) and the dominant (thick arrow) tumors, whose enhancement is more prominent in the intra-tumoral zone than in the peripheral zone. Another small nodule (long thin arrow) showed poor contrast enhancement. (D) Transverse fat-suppressed T1-weighted post-contrast image on the portal phase shows progressive enhancement at the peripheral and intra-tumoral zones of the dominant tumor (thick arrow) and the two small satellite tumors (long and short thin arrows). (E) and (F) Transverse diffusion-weighted image (b = 800, Fig 2E) and apparent diffusion coefficient map (Fig 2F) show different heterogeneous patterns relative to normal liver parenchyma, with various diffusion restrictions in the dominant (thick arrow) and the smaller tumors (long and short thin arrows).

**Fig 3:**
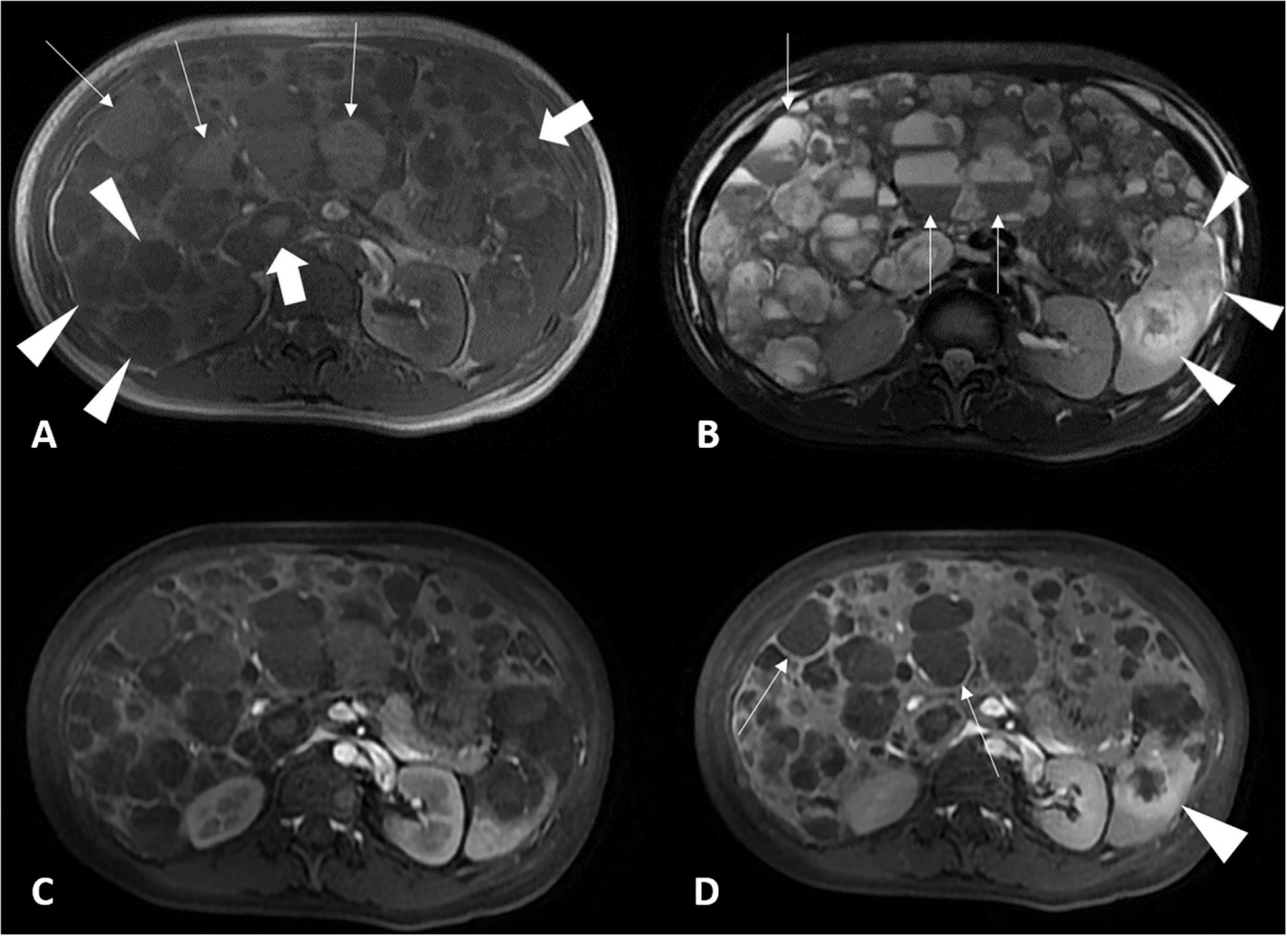
A 38-year-old women with primary hepatic angiosarcoma (case 2). (A) Transverse in-phase gradient-echo (TR/TE = 130/4.2) MR image shows multiple lesions display slight high (thin arrows), low (arrowheads) or both mixed signal intensity (thick arrows) at bilateral lobes of liver. (B) Transverse fat-suppressed T2-weighted fast spin-echo (TR/TE = 11428.6/91.312) MR image displays multiple lesions with mixed high and low signal intensity at bilateral lobes of liver. Some lesions show fluid-fluid levels (thin arrows). Splenic metastases are hyperintense on T2-weighted image (arrowheads), which are clearer than on in-phase MR image. (C) Transverse fat-suppressed T1-weighted (TR/TE = 100/1.8) post-contrast MR image on the arterial phase shows poor ring contrast enhancement in hepatic and splenic tumors. (D) Transverse fat-suppressed T1-weighted post-contrasted image on the portal phase displays ring enhancement at the peripheral zone of liver tumors (thin arrows) and splenic metastatic lesion (arrowhead).

PHA showed four tumor patterns on CT and MRI: single mass in three patients (3/22, 14%), multiple nodules with a dominant mass in 13 patients (59%), multiple nodules in three patients (14%), and diffuse infiltrating lesions in three patients (14%). Sixteen patients underwent variable abdominal CT scans: one patient with pre-contrast and post-contrast portal venous phase CT scans; 8 with pre-contrast and post-contrast triple-phase CT scans; 4 with pre- and post-contrast CT scans with arterial and portal venous phase; one with enhanced CT images on portal venous phase only; and two (case 6 and case 15) with pre-contrast CT abdominal images only because of their renal functional impairment to refuse intravenous iodine-based contrast medium with renal toxicity. The radiologists in this study identified tumor patterns of case 6 and case 15 as MNDM because the attenuation of multiple nodules (5 nodules for case 6; 13 nodules for case 15) and a dominant mass at bilateral hepatic lobes showed hypoattenuation with 36–45 HU greater than water attenuation (0–20 HU) of hepatic cysts (Fig 1). Case 15 also underwent abdominal MRI, which indicated his tumor pattern as MNDM. Pre-contrasted CT images showed the tumors of 11 patients (11/15, 73%, 6 survived ≤ 90 days, 5 > 90 days) to be heterogeneous low density with intra-tumoral high density, which indicated intra-tumoral hemorrhage (Fig 1A). In contrast, four PHA patients (4/15, 27%, 1 survived ≤ 90 days and 3 > 90 days) showed predominantly low density without intra-tumoral hyperdensity on pre-contrast CT. Twelve Of 13 patients received pre- and post-contrast triple-phase MRI protocols; 11 underwent 2D axial diffusion-weighted image (DWI) and apparent diffusion coefficient map (ADC); one underwent pre-contrast MRI protocol of abdomen only with DWI and ADC. Twelve patients with tumors (12/13, 92%, 4 survived ≤ 90 days, 8 > 90 days) showed predominant hypointensity with intra-tumoral hyperintense foci (Fig 2A) on pre-contrast axial in-phase, out-of-phase, and 3D axial and coronal FS T1WI, but predominant hyperintensity with intra-tumoral hypointense foci (Fig 2B) on pre-contrast 2D axial FS T2WI. The difference of intra-tumoral signal intensity differences on above-mentioned pre-contrast MR sequence mainly appeared in predominant or larger masses of PHA. The diffuse infiltrating lesions of case 18 surviving 36 days showed hypointensity on in phase, out of phase, FS T1WI and hyperintensity on FS T2WI, lacking an obvious focal point for different intensity areas. The MRI of case 2 showed multiple nodules with internal fluid-fluid level, which indicated intra-tumoral hemorrhage in multiple PHA and caused the lowest Hb (Hb = 4) (Fig 3). Total 18 of the 22 patients with abdominal CT scans and/or MRI had contrast enhanced images in a dynamic study including arterial and portal venous phase or triple phase, which showed heterogeneous, bizarre-shaped enhancement in the peripheral and internal zones of tumors on the arterial phase and progressive enhancement on portal venous phase in dominant or larger lesions (Fig 2C and 2D). Intra-tumoral contrast enhancement was often more prominent than peripheral contrast enhancement in the larger PHA tumors on arterial phase (16/18, 89%, 6 survived ≤ 90 days, 10 > 90 days) (Fig 2C and 2D). Only two patients (11%), case 2 (Fig 3) and case 11, showed higher ring enhancement (case 2, surviving 45 days) and bizarre-shaped enhancement (case 11, surviving 375 days) in the peripheral zones than in the intra-tumoral zones of PHA on the portal phase. Small PHA tumors showed poor to well contrast enhancement with a wide range on arterial and portal venous phase (Fig 2C and 2D). On DWI and the ADC map of the 11 patients, PHA showed a heterogeneous pattern relative to the normal liver parenchyma with various limited diffusion restrictions (Fig 2E and 2F).

No evidence of brain metastasis was noted in the three patients with brain CT. Due to the location of PHA in the liver, we used the AJCC 8^th^ TNM staging system for hepatocellular carcinoma to assess PHA [11]. Three patients were at stage I, 13 patients were at stage III, and 6 patients were at stage IV. No patients were at stage II in this study (Table 3).

As shown in Table 3, multiple nodules with a dominant mass were the most common tumor pattern of PHA on CT and/or MRI (11/22, 50%). Three (14%) of the 22 patients had multiple nodules. Eighteen (82%) of the 22 patients had tumors in the bilateral lobes of the liver, while the other four patients (18%) had tumors in the right lobe only. No patient had tumors in the left lobe of the liver only. Distant metastases were seen on images in six (27%) of 22 patients at presentation: lung metastases were detected in two patients (Fig 1B), splenic metastasis in one patient (Fig 3), pancreatic metastasis in one patient, splenic and lumber vertebral body metastases in one patient, and splenic and right pericardial metastases in one patient. Splenic metastases appeared in three (50%) of the six patients with metastases. Right portal vein thrombosis was noted in case 22 at stage III.

### Pathological diagnosis

**Fig 4:**
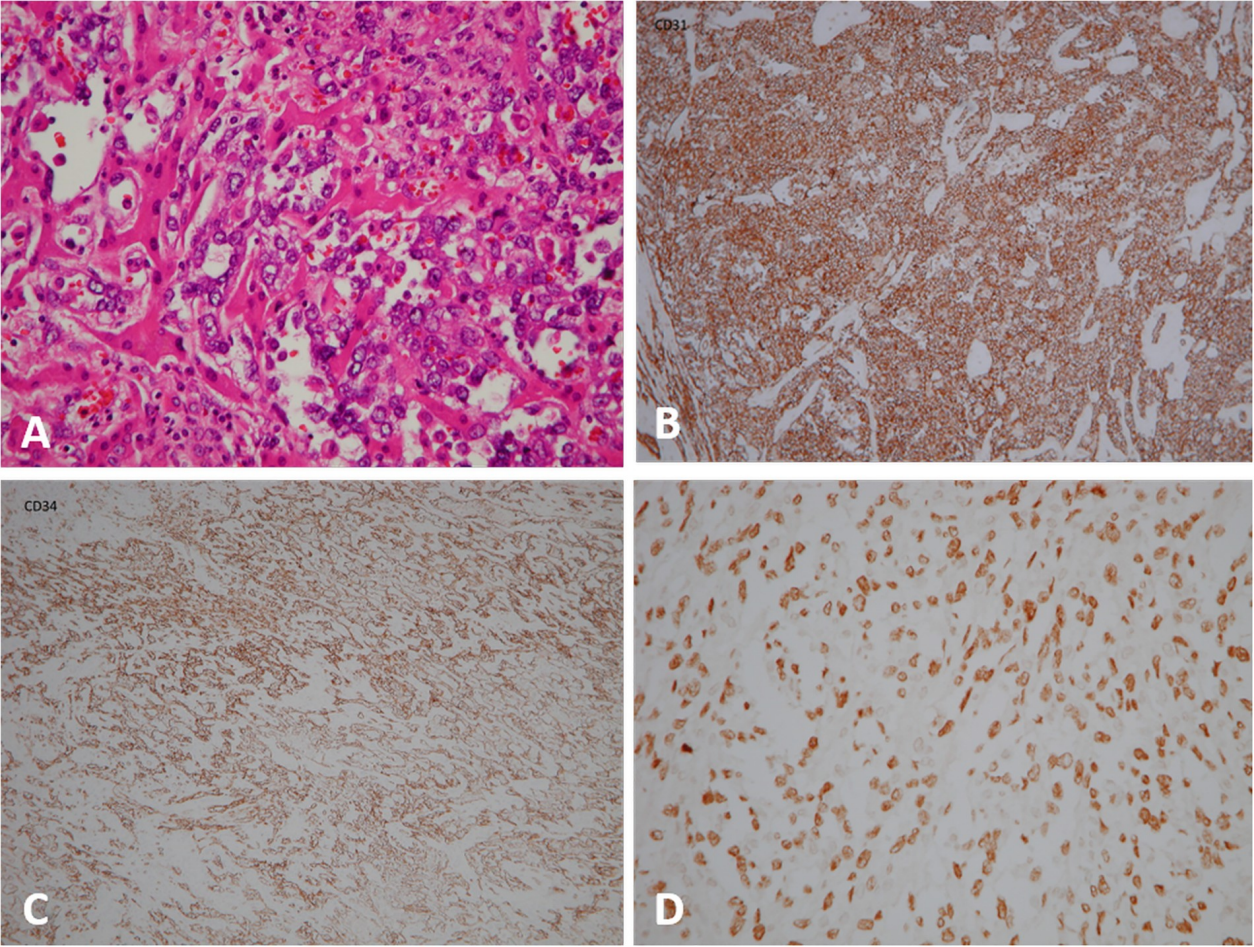
An 83-year-old woman with primary hepatic angiosarcoma (case 16). (A) Sections of the specimen show liver tissue with a poorly differentiated malignant tumor, which is composed of hyperchromatic and highly pleomorphic neoplastic cells arranged in a sheet-like pattern. The hepatic sinusoids are dilated and line the tumor cells (Hematoxylin-eosin, original magnification ×400). (B), (C) and (D) The tumor cells are immunoreactive for CD31 (x100), CD34 (x100), and ERG1 (x400).

A definitive pathological diagnosis of PHA confirmed by one experienced pathologist (AFYL) was based on an ultrasound-guided percutaneous liver biopsy in 17 patients, open liver biopsy in case 8 and case 16, surgical resection in case 4 and case 7, and trans-jugular vein liver biopsy in case 22. Pleomorphic tumoral endothelial cells with severe nuclear atypia and frequent mitoses grew along the dilated sinusoid, separating the surviving atrophic or hyperplastic hepatocytes (Fig 4A). In immunohistochemical stains (Table 3), the combination of two markers of CD31 and CD34 appeared in 14 (64%) of the 22 patients (Fig 4B and 4C). Stains for FLi-1, vWF, and ERG1-related antigens (Fig 4D) were also used in combination to diagnose PHA. Considering the aggressive behavior of PHA, the pathologist (AFYL) re-evaluated for the pathology of case 7 with an unrealistic overall survival of 2078 days and re-confirm the diagnosis of PHA.

### Treatment modality

As shown In Table 3, the treatment modality for PHA varied. The initial therapy for three stage I patients (cases 4, 5, and 7) was total tumor resection; all 3 cases had tumor recurrence during the follow-up period. Case 7 underwent a second bi-segmentectomy due to PHA recurrence. Eleven of 22 patients received intravenous or oral chemotherapy. Intravenous chemotherapy regimens included vinblastine sulfate, epirubicin, doxorubicin, bevacizumab, Ifosfamide, cisplastin, docetaxol, etoposide, pazopanib, paclitaxel, and gemcitabine. Oral chemotherapy included endoxan and thalidomide (case 5) and pazopanib (case 19). Case 15 and case 20 received intra-arterial brachytherapy with Yttrium-90 (Y-90) microsphere radioembolization. The chemotherapy used in trans-arterial chemoembolization (TACE) for PHA was adriamycin. The embolization material in trans-arterial embolization (TAE) and TACE consisted of lipiodol, gelfoam cubes, or microspheres (Embosphere). Seven (32%) of the 22 patients experienced initial or subsequent tumor rupture and underwent TACE or TAE: case 9 and case 11 during the initial admission; case 16 during the second admission; cases 1, 14, and 19 during the second admission after sono-guided biopsy during the initial admission; and case 22 after transjugular vein liver biopsy during the second admission. Case 16 underwent bi-segmentectomy of the liver after TACE. Five patients (cases 3, 6, 8, 12, and 17) with poor performance and/or distal metastases refused active treatment and received supportive care only, including nutritional support, psychosocial support, or pain control.

### Survival outcome

**Fig 5:**
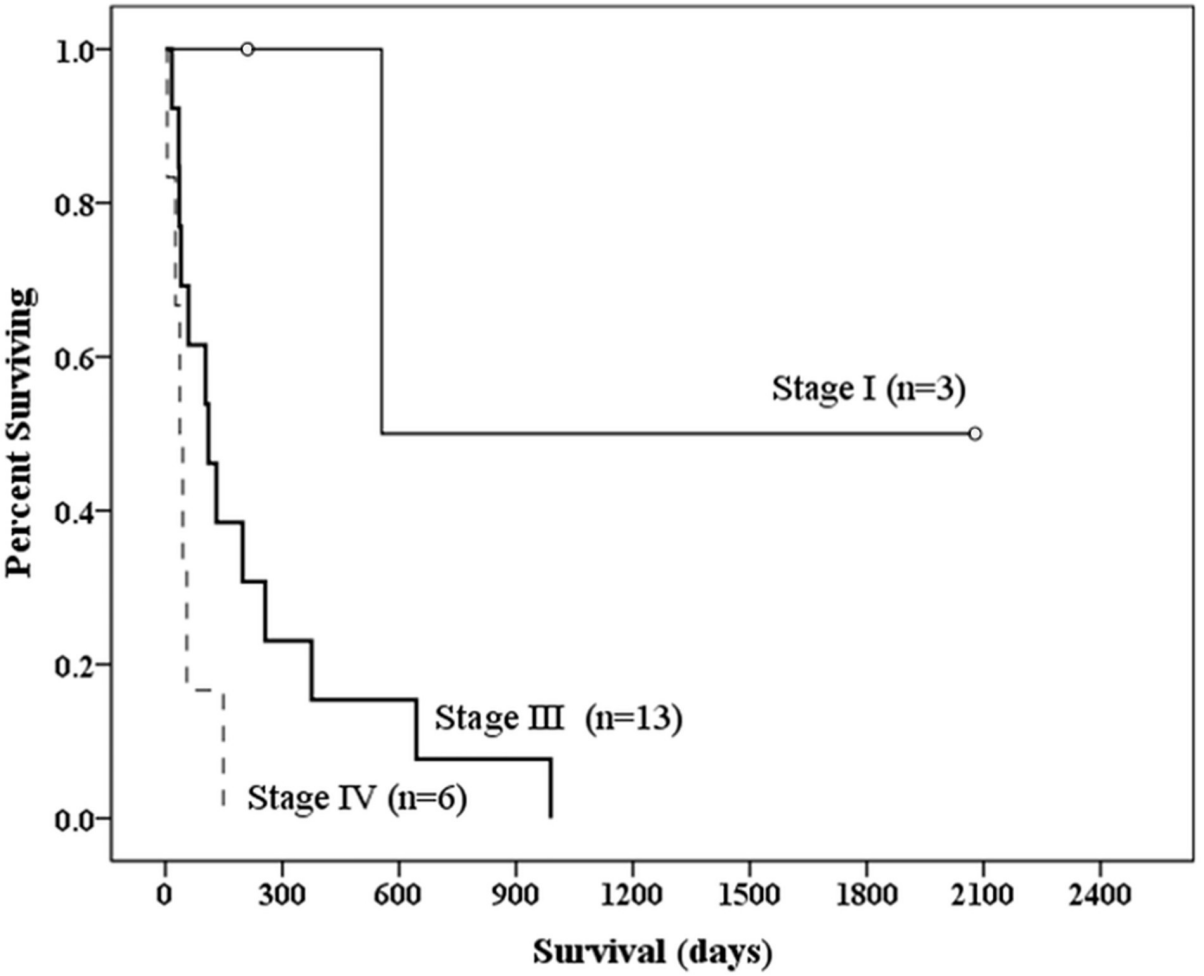
Survival analysis of 22 patients with primary hepatic angiosarcoma in relation to tumor-node-metastasis stage, using hepatocellular carcinoma staging system. Based on Kaplan-Meier survival curves, survival is significantly different between patients with stage I and those with stage IV disease (P = 0.011), between those with stage III and those with stage IV disease (P = 0.047) and between those with stage I and those with stage III disease (P = 0.044). Censored data: **o**.

Follow up started from the diagnosis of case 1 on November 12, 2009 and ended on February 23, 2018. The survival period was available in all cases. Twenty of the 22 patients died. The cause of death was sepsis in one patient, tumor rupture with hypovolemic shock and respiratory failure in three patients (cases 8, 11, and 14), and PHA progression causing hepatic and multi-organic failure in 16 patients. Overall survival (2078 days) of case 7 was censored data because he was still alive in the end (2018/2/23) of this study. Case 5 survived 211 days since diagnosis date on 2012/2/22 to the last clinic records with alive condition on 2012/9/19. Overall survival of case 5 was also censored data because we lost contact with case 5 and could not confirm if he was alive or dead from 2012/9/20 to the end (2018/2/23) of the study. The median follow-up period was 106.5 days (mean follow-up period, 279.7 days; range, 5–2,078 days). The 1-, 2-, 3-year survival rates were 23%, 9%, and 5%, respectively. The median survival of patients with stage I disease at diagnosis was 555 days (mean follow-up period, 948 days; range, 211–2,078 days). For patients with stage III disease at diagnosis, the median survival was 110 days (mean follow up, 230 days; range, 17–989 days). The median survival of patients with stage IV disease at diagnosis was 41 days (mean, 53 days; range, 5–149 days). No patients in the current study had stage II disease at diagnosis. Based on Kaplan-Meier survival curves (Fig 5), survival time differed significantly between patients with stage I and patients with stage IV disease (P = 0.011), between those with stage III and those with stage IV disease (P = 0.047), and between those with stage I and those with stage III disease (P = 0.044).

Table 4 summaries the demographic characteristics, medical history, and initial blood and laboratory tests of the 22 patients, compared by survival period (> 90 days vs ≤ 90 days). Higher levels of AST and TB, a lower level of ALB, longer PT and lower platelet count than the normal reference range were significantly associated with survival ≤ 90 days (all P < 0.05, Fisher’s exact test). The eight patients presenting with at least four abnormal levels of five significant tests (higher AST, TB, and PT, lower ALB and lower platelet count) all survived ≤ 90 days. Of the 10 patients who survived ≤ 90 days, eight (80%) had low platelet and abnormal levels of at least three of four markers (AST, TB, PT, or ALB). Five (62.5%) of these eight patients had stage III disease and three (37.5%) had stage IV disease. Of the 14 patients with less than four of these markers, 12 survived > 90 days and two (case 3 and case 6) survived ≤ 90 days. Patients with at least four abnormal test results had a significantly short survival period (≤ 90 days) than those with fewer abnormal results (P = 0.0001, Fisher’s exact test). WBC, Hb, ALT, AFP, CA-199, and CEA did not differ significantly between the two groups (P > 0.05, Fisher’s exact test).

**Table 4:** Initial clinical and laboratory data between two groups of primary liver angiosarcoma (10 cases with survival ≤ 90 days and 12 cases with survival > 90 days).

Clinical and Lab data	10 Cases with survival ≤ 90 days	12 Cases with survival > 90 days	P value
Age (Median±SD)	65.5±13.0	71.5±11.2	0.936^#^
Male/female	6/4	9/3	0.6517*
DM (+/-)	3/7	7/5	0.2305*
Hypertension (+/-)	3/7	7/5	0.2305*
Smoking (+/-)	2/8	4/8	0.6462*
Drinking (+/-)	2/8	4/8	0.6462*
Virus hepatitis (+/-)	1/9	2/10	1.0000*
Liver cirrhosis (+/-)	1/9	3/9	0.5940*
Previous malignancy (+/-)	1/9	3/9	0.5940*
Liver, Bilateral/Rt lobe	10/0	8/4	0.0964*
Tumor rupture at initial presentation (+/-)	0/10	2/10	0.4805*
WBC (Ab/N)	3/7	2/10	0.6241*
Hb (L/N)	10/0	9/3	0.2208*
PLT (L/N)	10/0	4/8	0.0017*
ALT (H/N)	5/5	2/10	0.1718*
AST (H/N)	7/3	1/11	0.0062*
ALB (L/N)	7/1	4/8	0.0281*
TB (H/N)	7/2	1/11	0.0022*
PT (H/N)	7/3	1/11	0.0062*
AFP (H/N)	0/7	0/12	1*
CEA (H/N)	0/7	1/9	1*
CA-199 (H/N)	1/6	0/11	0.3889*

^#^Mann-Whitney U test;

*Fisher exact test;

SD, standard deviation; DM, Diabetes mellitus; WBC, white blood cell; Hb, hemoglobin; PLT, Platelet; ALT, alanine transaminase; AST, aspartate transaminase; ALB, albumin; TB, total bilirubin; PT, prothrombin time; AFP, alpha-fetoprotein; CEA, carcinoembryonic antigen; CA-199, carbohydrate antigen; +/-, cases with positive result/cases with negative result; Ab/N, cases with abnormal data/cases within normal data; H/N, cases with higher data than normal/cases within normal data; L/N, cases with lower data than normal/cases within normal data.

Not all the 22 patients received CT or MRI. The study found no any statistically significant correlation between a short survival (≤ 90 days) and present following imaging features, including intra-tumoral high density hemorrhage on pre-contrast CT, intra-tumoral heterogenous signal intensity on pre-contrast MRI (in phase, out of phase, FS T1WI, FS T2WI) and prominent bizarre-shaped enhancement in larger tumors on post-contrast arterial-phase CT and MRI (P = 0.5692, 0.3846, and 1, respectively, Fisher’s exact test).

Moreover, the study compared the initial clinical and laboratory data of patients with different tumor patterns, including SM vs MNDM, SM vs MN, SM vs DIL, MNDM vs MN, MNDM vs DIL and MN vs DIL, and found no statistically significant difference between those two groups (all P > 0.05, Fisher’s exact test) except SM group showed more common tumor location in right lobe of liver than MNDM group (P = 0.0071, Fisher’s exact test).

**Fig 6:**
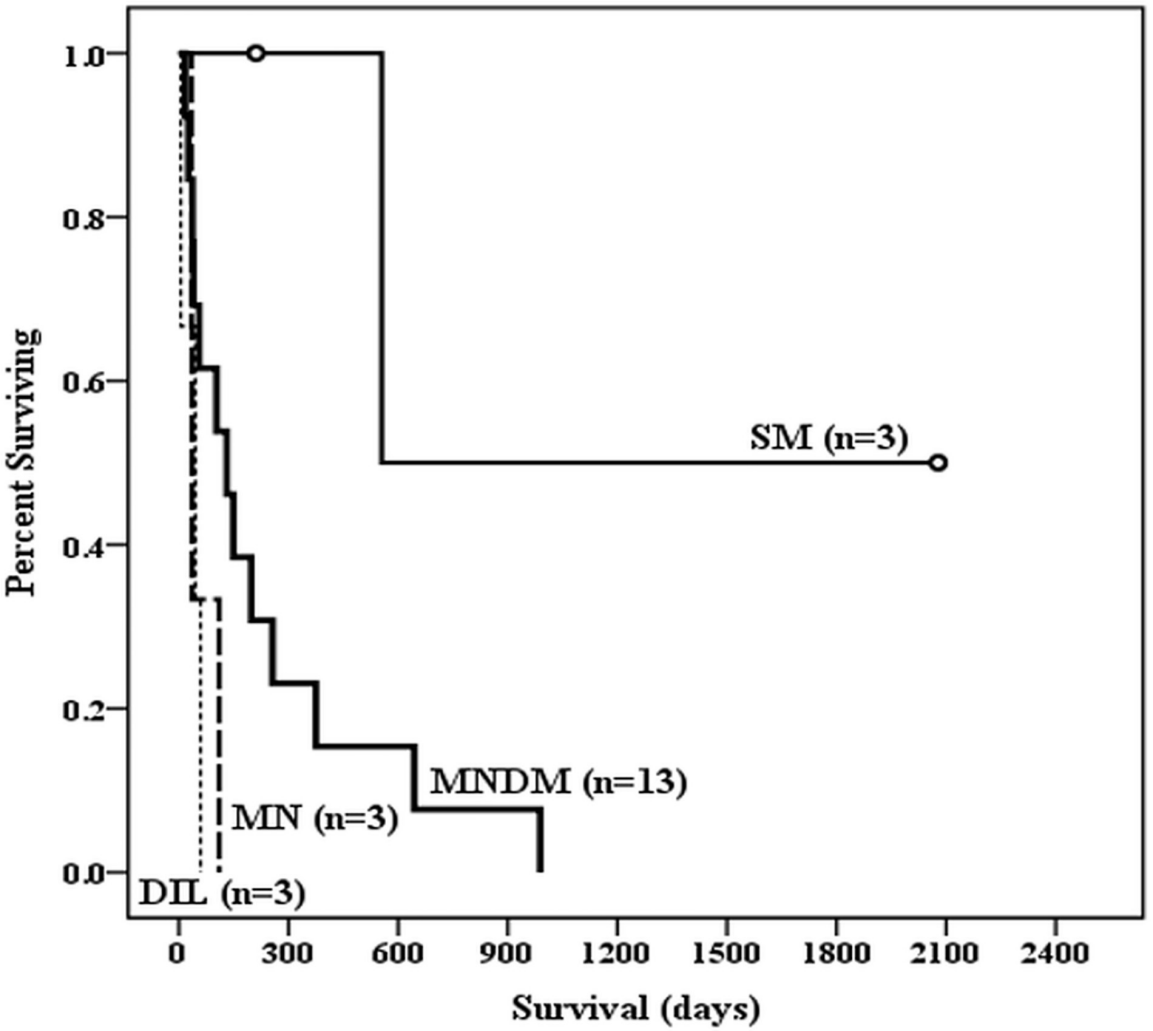
Survival analysis of the 22 patients with primary hepatic angiosarcoma in relation to tumor patterns in liver. Based on Kaplan-Meier survival curves, survival is significantly different between patients with single mass (SM) and those with multiple nodules with a dominant mass (MNDM) (P = 0.044), SM vs multiple nodules (MN) (P = 0.025) and SM vs diffuse infiltrating lesions (DIL) (P = 0.025). Survival is not significantly different between those with MNDM vs MN, MNDM vs DIL, and MN vs DIL (P = 0.068, 0.694, and 0.093, respectively). Censored data: o.

Nevertheless, the study compared the survival of the patients with different tumor patterns of PHA based on Kaplan-Meier survival curves (Fig 6) and found much better survival in patients with SM (SM vs MNDM, P = 0.044; SM vs MN, P = 0.025, and SM vs DIL, P = 0.025) but no significant survival difference between those with MNDM vs MN, MNDM vs DIL, and MN vs DIL (all P > 0.05).

## Discussion

PHA is a rare, highly aggressive neoplasm, with a median survival of less than 180 days from the time of diagnosis [16, 17]. In contrast, the median follow-up period in our study was 106.5 days, less than 180 days. The current study evaluated the association between initial clinical-radiological features and patient’s prognosis. Those who survived > 90 days had significantly different serum levels of AST, ALB, TB, and PT, and platelet count than those who survived ≤ 90 days (P < 0.05). OS was much better in patients with SM than in those with MNDM, MN or DIL (SM vs MNDM, P = 0.044; SM vs MN, P = 0.025, and SM vs DIL, P = 0.025). Furthermore, OS of the studied 22 patients in relation to TNM stage of PHA determined by preliminary imaging showed significant differences between stage I versus stage III (P = 0.044), stage I versus stage IV (P = 0.011), and stage III versus IV (P = 0.047).

Thrombocytopenia, which was noted in 14 (64%) of 22 patients and anemia (19/22, 86%) were relatively common in our study and may be related to the trapping of platelets in the poorly organized neoplastic vessels of PHA and the consumption of clotting factors [4, 16], which could induce an initial and subsequent spontaneous rupture of PHA and lead to intraabdominal bleeding. While a biopsy was important for diagnosis of PHA, percutaneous liver biopsy in patients with PHA was dangerous because of the great tendency toward hemorrhage [20]. Many studies recommended open biopsy or fine needle aspiration for PHA instead of percutaneous liver biopsy [20].

Kalva et al. reported that Child-Pugh grading and preliminary imaging for staging offered essential prognostic information for treatment of liver tumors [21]. The assessed factors of Child-Pugh grading were TB level, serum ALB, PT or international normalized ratio, degree of ascites and degree of hepatic encephalopathy. Findings of our study showed that all eight (80%) of 10 patients who survived ≤ 90 days had at least 4 of 5 abnormal factors (AST, ALB, TB, PT and platelet count), which were consistent with the results of Kalva et al. AST and ALT are abundant enzymes in hepatocytes: AST is found in both cytosol and mitochondria, but ALT only in cytosol [22]. ALB and prothrombin reflect the synthetic function of the liver and TB represents the elimination function of the liver [23]. In our study, advanced PHA usually occupies a large volume of the liver, causing injury or death to hepatocytes, which may explain the elevation of AST and TB, the decline of ALB, and the prolongation of PT [22]. Five (62.5%) of these eight patients had stage III disease and three (37.5%) had stage IV disease, which indicated that five initial laboratory tests (AST, ALB, TB, PT, platelet count) could provide effective prognostic information, even without imaging information.

Some studies showed that patients with PAH have liver function tests that show only mild hyperbilirubinemia and slightly elevated AST/ALT [4]. However, AST, while not included in Child-Pugh grading, was an important prognostic indicator of PHA in this study. The test profile of ALT and Hb were not statistically different between the two groups (survival ≤ 90 days versus > 90 days, Table 4). The reason might be the small sample size of the current study. In addition to liver biochemistry and Child-Pugh classification, liver functional reserve can be assessed by measuring the indocyanine green retention rate at 15 minutes [24].

In our study, 19 (86%) of the 22 patients with PHA had multiple tumor lesions in the liver on CT and MRI. Numerous hepatic lesions usually indicate a malignant nature [1]. The primary features of multiple tumor lesions of PHA were hypodense findings on pre-contrast CT; hypointense findings on T1WI; and hyperintensity on FS T2WI on pre-contrast MRI compared with normal liver parenchyma [1, 16]. Intra-tumoral hemorrhage of PHA in various sizes, which was usually absent in cholangiocarcinoma, was present as high density on pre-contrast CT, hyperintensity on pre-contrast T1WI, and hypointensity on pre-contrast FS T2WI [1, 25]. Obvious intralesional hemorrhage even displayed a fluid-fluid level in PHA, as in our case 2. Moreover, on pre-contrast FS T2WI, fibrosis in PHA showed as hypointensity and necrosis showed as hyperintensity [1]. On the arterial phase of dynamic enhanced CT and MRI, enhancement in bizarre shapes mainly in the intra-tumoral regions of PHA could be distinguished from the nodular enhancement on peripheral zones in benign hemangiomas [1, 16]. On the portal venous phase, the predominant PHA mass displayed heterogeneously progressive but incomplete enhancement, which could help differentiate PHA from hepatocellular carcinoma and liver metastases, even though this pattern also appeared in cholangiocarcinoma. Small nodules showed homogeneous contrast fill-in on portal venous phase CT or MRI. The varied contrast enhancements reflected differences in the microvascular structures within PHA [1, 16]. On DWI, dominant masses displayed heterogeneous hyperintensity mixed with hypointense structures, but most of the small PHA nodules showed homogeneous hyperintensity only [16]. The DWI of PHA showed a wide range of inter-tumoral and intra-tumoral ADC values (range 0.57 to 2.41x10^−3^ mm^2^/s, mean 1.37x10^−3^ mm^2^/s). In the dominant masses of PHA, high ADC values (> 2x 10^−3^ mm^2^/s) indicated relatively unrestricted diffusion, reflecting cystic necrosis in pathology; intermediate values (1.5–2x10^−3^ mm^2^/s) usually exhibited dilated sinusoidal or cavernous spaces; and low values corresponded to hematoma [16].

Pathological stage (surgical stage) determines the exact extent of a patient’s cancer by examination of the removed operative tissue to decide the best therapeutic plan for a patient [26]. However, in the current study, we used preliminary imaging stage for hepatocellular carcinoma to evaluate patients instead of the pathological stage of the AJCC TNM staging system for soft tissue sarcoma for two reasons. One reason was that the primary angiosarcoma was in the liver, and the other was the majority of patients (18/22, 82%), with highly aggressive multiple PHA in bilateral lobes of the liver, underwent chemotherapy or conservative treatment without operation. Preliminary imaging stage was based on the patient’s initial images, which were performed routinely or according to symptoms and signs or physical examination before the first formal treatment with a pathological proof. This study showed significant difference in OS of patients at stage I from patients at stage III, as well as of stage III from stage IV, and of stage I from stage IV (P < 0.05) disease, which proved that the preliminary imaging stage of PHA using the hepatocellular carcinoma staging system could provide effective prognostic information for our patients. However, some PHA patients might also need brain CT, brain MRI, or chest CT to improve the evaluate of their imaging stage before the first formal treatment with a pathological proof.

The standard treatment guidelines for PHA have not been established because the tumor is extremely rare and aggressive [11, 20]. Complete surgical resection is the only therapeutic modality that can provide a potential cure if PHA is resectable [11, 27]. In the current study, cases 4, 5, and 7 underwent complete surgical resection of PHA and got long-term survival, although all three later had tumor recurrence in the liver. Patients with unresectable or recurrent hepatic angiosarcomas might receive palliative treatments, such as trans-arterial embolization, radiation therapy, radiofrequency ablation, percutaneous ethanol injection, chemotherapy, or combined therapy [7]. These palliative treatments were associated with various outcomes in previous studies [11]. Most studies showed that chemotherapy and radiation therapy had no significant effect on survival [11, 20, 28]. Liver transplant for patients with PHA should not be recommended to patients with a high tumor recurrence rate and a median survival time of less than seven months after liver transplant [20]. Our patients underwent various treatment modalities, including supportive care, palliative treatment or combined therapies, depending on each patient’s general condition and hepatic function.

Two patients in the current study received intra-arterial brachytherapy with Y-90 microsphere radioembolization, an increasingly used treatment for unresectable primary hepatic tumors (hepatocellular carcinoma) and liver metastases of colorectal and neuroendocrine tumors [21, 29]. One study showed no significant difference in median OS between patients with primary and metastatic hepatic angiosarcoma undergoing embolization, chemoembolization or Y-90 radioembolization (P = 0.76) [30]. Therefore, further evaluation of Y-90 radioembolization to treat PHA is necessary.

There are several limitations to this study. First, this retrospective chart review had a small number of patients. Second, the initial clinical information before the first formal treatment with a pathological proof was not always complete in the chart, a weakness of the retrospective nature of the study. Laboratory data with missing values included serum ALB, TB, AFP, CEA, and CA-199. Because risk factors were absent in the charts, we were unable to evaluate patients’ exposure history of risk factors for PHA. Third, the definitive TMN stage of patients with PHA was hard to evaluate because most of these patients had multiple or diffuse infiltrating tumors and could not undergo an operation. A pretherapeutic decision of metastatic lymphadenopathy depended on the lesion size on the abdominal CT or MRI. Finally, preliminary images for staging before the first formal treatment with a pathological proof might not have been sufficiently rigorous, although all patients received chest x-ray and either abdominal CT or MRI or both. Patients did not routinely receive chest CT, brain CT/MRI, whole body bone scan, or whole-body PET/CT unless the clinician suspected the presence of a metastatic lesion.

### Conclusion

PHA should be the highly suspected diagnosis when CT and/or MRI shows: multiple hepatic tumors present with a dominant mass containing some internal hyperdense foci on pre-contrasted CT scans; intra-tumoral different signal intensity on pre-contrast in phase, out of phase, FS T1W and FS T2W MRI; and heterogeneous contrast enhancement with bizarre shapes mainly in intra-tumor zones of larger tumors on arterial phase and progressive enhancement on portal phase of dynamic post-contrasted CT and/or MRI. Clinicians should perform liver biopsy very carefully or use alternate methods to avoid postoperative intraabdominal bleeding. Our study also showed that initial abnormal data with low platelet count, high AST, low ALB, high TB, and prolonged PT were significantly associated with patient survival ≤ 90 days. Survival was much better in patients with SM than in those with other tumor patterns (SM vs MNDM, P = 0.044; SM vs MN, P = 0.025, and SM vs DIL, P = 0.025). The estimated clinical stage of patients with PHA using preliminary images based on the 8th AJCC staging of hepatocellular carcinoma enabled doctors to predict patient survival before treatment (stage I vs stage III, stage III vs stage IV, and stage I vs stage IV, all P < 0.05). Patients having PHA with more normal results of five laboratory tests (platelet count, AST, TB, ALB, and PT) or early stage disease estimated by preliminary images would present a better prognosis despite the treatment used. Because of the small number of patients in the current study, further prospective or meta-analytic study of the prognostic factors associated with PHA is needed.

## Abbreviations

ADC: apparent diffusion coefficient map
AFP: alpha-fetoprotein
AJCC: American Joint Committee on Cancer
ALB: albumin
ALT: alanine aminotransferase
AST: aspartate aminotransferase
CA 19–9: carbohydrate antigen19–9
CEA: carcinoembryonic antigen
CM: contrast medium
CT: computed tomography
DIL: diffuse infiltrating lesions
DWI: diffusion-weighted image
FOV: field of view
Gd: gadolinium
GRE: gradient-echo
Hb: hemoglobin
I: iodine
MDCT: multiple detector computed tomography
MN: multiple nodules
MNDM: multiple nodules with a dominant mass
MRI: magnetic resonance imaging
OS: overall survival
PET: positron emission tomography
PHA: primary hepatic angiosarcoma
PT: prothrombin time
SM: single mass
T1WI: T1-weighted MR image
T2WI: T2-weighted MR image
TACE: trans-arterial chemoembolization
TAE: trans-arterial embolization
TB: total bilirubin
TE: time of echo
TNM: tumor-node-metastasis
TR: time of repetition
WBC: white blood cell
Y-90: Yttrium-90
